# Effect of Tranexamic Acid to Reduce Postoperative Hematoma in Reduction Mammaplasty: A Retrospective Analysis of 656 Patients

**DOI:** 10.1007/s00266-026-06101-w

**Published:** 2026-06-15

**Authors:** Joni Ilmanen, Salvatore Giordano

**Affiliations:** 1 Department of Plastic and General Surgery, Turku University Hospital, University of Turku, OS 299, PL 52, 20521 Turku, Finland; 2 Department of Surgery, Satasairaala Hospital, Pori, Finland; 3 University of Turku, Turku, Finland

**Keywords:** Tranexamic acid, Reduction mammoplasty, Hematoma, Perioperative bleeding, Postoperative complications

## Abstract

**Background:**

Postoperative hematoma requiring reoperation is a clinically significant complication in breast surgery. Although tranexamic acid (TXA) is widely used in other surgical specialties to reduce bleeding, its routine use in breast surgery remains limited due to insufficient evidence.

**Aims and Objectives:**

This study aimed to evaluate whether intraoperative TXA reduces the incidence of hematoma following reduction mammaplasty.

**Materials and Methods:**

We conducted a retrospective, single-center cohort study including 656 consecutive patients who underwent reduction mammaplasty between 2019 and 2024. TXA was administered intravenously (n = 124, 43%), topically (n = 124, 43%), or via both routes (n = 39, 14%) according to surgeon preference. Patients exposed to TXA were compared with those unexposed.

**Results:**

TXA use was associated with a significantly lower rate of postoperative hematomas requiring reoperation (2.1% vs. 5.1%; OR 2.3, 95% CI: 1.1–5.0; *p* = 0.042). Operative time was longer in the TXA group (133 vs. 122 minutes; *p* < 0.001), and patients receiving TXA had higher intraoperative blood loss and breast resection weights. No significant differences were observed in overall complications or 30-day reoperation rates, although superficial infections were more frequent in the non-TXA group (3.8% vs. 0.7%; *p* = 0.022). Multivariable analysis suggested a trend toward TXA as an independent protective factor. No TXA-related adverse events were reported.

**Conclusions:**

Intraoperative TXA in reduction mammaplasty was associated with a more than twofold reduction in postoperative hematomas. As a simple and low-risk intervention, TXA may reduce the need for reoperation. Large randomized controlled trials are warranted to confirm these findings and guide definitive clinical recommendations.

**Level of Evidence III:**

This journal requires that authors assign a level of evidence to each article. For a full description of these Evidence-Based Medicine ratings, please refer to the Table of Contents or the online Instructions to Authors www.springer.com/00266.

## Introduction

Reduction mammaplasty, or breast reduction, is a commonly performed procedure in plastic surgery indicated for the treatment of macromastia, a condition characterized by hypertrophy of glandular breast tissue [1, 2]. Macromastia is associated with a spectrum of physical symptoms, including back, neck, and shoulder pain, headaches, intertriginous rashes, and pruritus, as well as psychological distress, such as low self-esteem, depression, and negative body image [3–5]. Multiple studies have demonstrated that reduction mammaplasty effectively alleviates both the physical and psychological sequelae of macromastia [4–9].

Reduction mammaplasty is generally considered a safe surgical intervention, with reported postoperative patient satisfaction rates reaching up to 97% [9–11]. Nonetheless, as with any surgical procedure, it carries a risk of complications, including seroma and hematoma formation, wound dehiscence, tissue necrosis, delayed wound healing, and surgical site infections [11–13]. Although most postoperative complications are minor, hematoma formation represents a clinically significant concern, as it may necessitate reoperation, delay wound healing, increase the risk of infection, and adversely affect aesthetic outcomes [3, 11, 12, 14]. The reported incidence of postoperative hematoma following reduction mammaplasty ranges from 0.3 to 7% [3, 12, 15, 16].

Tranexamic acid (TXA), developed in the 1960s, is a well-established antifibrinolytic agent that inhibits the conversion of plasminogen to plasmin, thereby preventing fibrin degradation. In addition to its hemostatic properties, TXA may exert anti-inflammatory effects and enhance platelet function [11, 17–20]. Originally indicated for heavy menstrual bleeding and hereditary bleeding disorders, its use has expanded to surgical, traumatic, and postpartum hemorrhage [21]. TXA is now widely employed across surgical specialties, including cardiothoracic and orthopedic surgery, to reduce blood loss and perioperative complications [18, 22–24].

In reduction mammaplasty, TXA administration, commonly via intravenous or topical routes [11, 22], has been investigated to mitigate complications, particularly postoperative hematoma [2, 3, 11, 12, 15, 18, 22, 25–29]. Hematomas may lead to infection, wound dehiscence, delayed healing, and tissue necrosis, resulting in additional procedures, prolonged hospitalization, and increased patient morbidity [10, 13, 30]. Although several meta-analyses support the safety and efficacy of TXA in breast surgery in general [3, 12, 15, 29], and one recent meta-analysis has demonstrated reduced hematoma formation specifically in reduction mammaplasty [15], the evidence remains limited due to small sample sizes and occasional conflicting results across studies [27]. Existing studies are heterogeneous in methodology and outcomes, limiting definitive conclusions regarding the effectiveness of TXA in reduction mammaplasty and underscoring the need for more robust, procedure-specific evidence. Consequently, TXA use is not yet routine in reduction mammaplasty, highlighting the need for further investigation to optimize its administration and improve surgical outcomes.

This study aimed to evaluate whether intraoperative administration of tranexamic acid (TXA) reduces the incidence of postoperative hematoma following reduction mammaplasty. We hypothesized that TXA use would decrease both hematoma formation and the subsequent need for reoperation.

## Materials and Methods

We conducted a retrospective cohort study of patients with breast hypertrophy who underwent reduction mammaplasty (RM) at a single academic institution. All consecutive patients undergoing primary bilateral RM between January 2019 and December 2024 were included. Data were extracted from electronic medical records, including preoperative assessments and postoperative follow-up from outpatient visits. The study was approved by the local institutional review board (protocol T145/2024; protocol code T104/2020; approved April 13, 2024) and conducted in accordance with the ethical principles of the Declaration of Helsinki. Due to the retrospective design and anonymization of patient data, the requirement for individual informed consent was waived.

Inclusion criteria comprised female patients aged ≥ 18 years undergoing primary bilateral RM with a minimum follow-up of three months. Exclusion criteria included male sex, age < 18 years, unilateral procedures, mastopexy, secondary breast reduction, and resections performed for oncological indications. During the study period, national eligibility criteria for RM required cervical syndrome unresponsive to physiotherapy, a jugulum–nipple distance > 27 cm, and a body mass index (BMI) ≤ 30 kg/m^2^. Collected covariates included age, BMI, smoking status, diabetes, hypertension, use of anticoagulant or antiplatelet medication, operative time, estimated blood loss, and resection weight.

Patients were categorized into two groups based on intraoperative tranexamic acid (TXA) exposure: those who received TXA (topical and/or intravenous) and those who did not. Most procedures were performed using Wise-pattern markings with a superomedial pedicle. During the study period, 21 board-certified plastic surgeons performed the procedures. TXA administration was not standardized and was based on surgeon preference and intraoperative judgment.

Intravenous TXA (1000 mg) was administered within 60 minutes prior to incision, typically during anesthesia induction. Topical TXA (1000 mg diluted in saline to a concentration of 20–25 mg/mL) was applied to the surgical field prior to wound closure and allowed to dwell for several minutes before suction evacuation. In some cases, both intravenous and topical TXA were used.

The primary outcome was postoperative hematoma requiring surgical intervention. Reoperation was defined as any return to the operating room for hematoma evacuation at the surgical site following RM.

Secondary outcomes included intraoperative blood loss, operative time, length of hospital stay, overall postoperative complications, wound-healing disorders, complications during follow-up, and late reoperation rates. Complications were defined as wound dehiscence, fat necrosis, cellulitis, abscess formation, nipple necrosis, and systemic medical complications.

Hematomas and seromas were defined as subcutaneous collections of blood or serous fluid requiring percutaneous aspiration or surgical evacuation. Intraoperative blood loss was quantified as the total suction canister volume, including fluid expressed from surgical swabs. Wound dehiscence was defined as full-thickness separation of wound edges > 2 cm, with or without infection. Skin necrosis was defined as a clearly demarcated necrotic area > 1 cm, and fat necrosis as a palpable mass ≥ 1 cm persisting for more than three months postoperatively. Surgical site infections were defined as localized cellulitis or abscess requiring oral or intravenous antibiotics, with or without operative management. Nipple necrosis was classified as partial or complete ischemia/necrosis of the nipple–areolar complex. Infections were recorded when antibiotic therapy was initiated, and all prescribed medications were verified through the centralized Finnish national prescription database, enabling identification of cases even if patients did not contact the surgical unit directly.

Systemic and anesthetic complications included deep vein thrombosis, pulmonary embolism, and other perioperative adverse events. Postoperative follow-up evaluations were conducted by plastic surgeons at approximately 1, 3, and 6–9 months.

### Statistical Analysis

Continuous variables were summarized as means with standard deviations (SD), whereas categorical variables were presented as frequencies and percentages. The distribution of continuous variables was assessed for normality using histograms, skewness and kurtosis measures, and the Kolmogorov–Smirnov test. Between-group comparisons (TXA vs. no TXA) were performed using Student’s *t*-test for normally distributed continuous variables. Categorical variables were compared using Pearson’s chi-square test or Fisher’s exact test, as appropriate.

Multivariable logistic regression analysis was performed to identify independent predictors of postoperative hematoma and wound-healing complications. The primary model assessed the association between TXA exposure and hematoma formation, with results reported as adjusted odds ratios (ORs) with corresponding 95% confidence intervals (CIs). Covariates entered into the model included TXA exposure, BMI, smoking status, diabetes, hypertension, anticoagulant or antiplatelet use, resection weight, operative time, and estimated blood loss. Model fit was evaluated using the Hosmer–Lemeshow goodness-of-fit test, which indicated an adequate fit (*p* = 0.114).

All statistical tests were two-sided, and a p-value ≤ 0.05 was considered statistically significant. Statistical analyses were performed using IBM SPSS Statistics, version 31.0 (IBM Corp., Armonk, NY, USA).

## Results

Between January 2019 and December 2024, a total of 656 consecutive reduction mammaplasty procedures were performed. Of these, 287 patients (44%) received intraoperative TXA, while 369 (56%) did not. Among patients receiving TXA, administration was intravenous in 124 cases (43%), topical in 124 cases (43%), and combined intravenous and topical in 39 cases (14%), according to surgeon preference. For the primary analysis, patients were categorized into two groups: TXA-exposed (n = 287) and unexposed (n = 369).

Baseline patient characteristics (Table 1) were comparable between groups, with no significant differences in the analysed variables. Follow-up duration was significantly longer in the TXA-unexposed group (*p* < 0.001), whereas mean operative time was significantly longer in the TXA-exposed group (*p* < 0.001). Patients receiving TXA had a greater mean breast resection weight, reaching statistical significance for the left breast (*p* = 0.034), and higher intraoperative blood loss compared with the unexposed group (263.8 mL vs. 229.5 mL; *p* = 0.014; Table 2).

**Table 1:** Patient demographics at the time of the study

	Exposed to TXA (n = 287)	Unexposed to TXA (n = 369)	*p*-value
Age (mean ± SD)	42.2 ± 15.1	43.6 ± 15.0	0.231
Mean BMI (kg/m^2^)	26.8 ± 2.2	26.8 ± 2.4	0.995
Any comorbidity	149 (51.9%)	173 (46.9%)	0.201
Diabetics	8 (2.8%)	2 (2.2%)	0.610
Hypertension	36 (12.5%)	43 (11.7%)	0.728
Pulmonary disease	22 (7.7%)	29 (7.9%)	0.927
Depression	38 (13.2%)	53 (14.4%)	0.680
Smokers	38 (13.2%)	44 (11.9%)	0.613
Herbal supplement	14 (4.9%)	31 (8.4%)	0.087
Follow-up (months)	3.2 ± 2.4	5.0 ± 5.6	<0.001

**Table 2:** Comparison of perioperative parameters between the two patient groups

	Exposed to TXA (n = 287)	Unexposed to TXA (n = 369)	*p*-value
Operative time (min, mean ± SD)	133.1 ± 33.7	122.5 ± 32.8	< 0.001
Resection weight from right breast (g, mean ± SD)	606.9 ± 267.3	573.2 ± 242.7	0.091
Resection weight from left breast (g, mean ± SD)	619.1 ± 263.5	576.8 ± 244.7	0.034
Blood loss (ml, mean ± SD)	263.8 ± 203.6	229.5 ± 152.9	0.014
Hospital stay (days, mean ± SD)	0.53 ± 0.63	0.53 ± 0.72	0.904

TXA use was associated with a significantly lower incidence of postoperative hematomas requiring reoperation (2.1% vs. 5.1%; OR 2.3, 95% CI: 1.1–5.0; *p* = 0.042; Table 3). Most hematomas in both groups occurred within the first 48 postoperative hours. No delayed clustering (postoperative days 5–7) was observed in the TXA group compared with the non-TXA group.

**Table 3:** Postoperative complications during follow-up

	Exposed to TXA (n=287)	Unexposed to TXA (n=369)	*p*-value
Patients with complications	21 (7.3%)	35 (9.5%)	0.324
*Complications*			
Superficial wound infection (received antibiotics < 30 days)	2 (0.7%)	14 (3.8%)	0.022
Deep wound infection (revision in local anaesthetics)	21 (7.3%)	17 (4.6%)	0.104
Wound dehiscence (T-junction mostly, need for revision -local/general)	12 (4.2%)	12 (3.3%)	0.529
Fat necrosis (need for operation)	2 (0.7%)	6 (1.6%)	0.282
Hematoma (need for operation)	6 (2.1%)	19 (5.1%)	0.042
Nipple necrosis (partial/total, requiring extra follow-up/procedure)	5 (2.1%)	7 (3.1%)	0.883
Reoperation <30 days	2 (0.7%)	19 (5.1%)	0.241
Reoperation at follow up, more than 30 days post operatively	1 (0.3%)	11 (3.0%)	0.017
Reoperation for dog-ear	4 (1.4%)	13 (3.5%)	0.249

No significant differences were observed in overall postoperative complication rates between the two groups. Superficial infections occurred more frequently in patients unexposed to TXA (3.8% vs. 0.7%; *p* = 0.022), while no other significant differences in postoperative complications were identified. Reoperation rates within 30 days were similar between groups; however, late reoperations were significantly less frequent in the TXA group (0.3% vs. 3.0%; *p* = 0.017; Table 3).

Multivariable analysis did not identify any independent risk factors for hematoma formation (Table 4); however, TXA exposure demonstrated a trend toward a protective effect. No adverse events attributable to TXA were reported or recorded in the institutional surgical registry.

**Table 4:** Multivariable logistic regression analysis of independent risk factors for hematoma formation, including adjusted odds ratios for TXA use

	Odd ratios	95% Confidence interval	*p*-value
BMI	0.95	0.71–1.28	0.748
Smoking	1.54	0.30–7.82	0.601
Operative time	0.99	0.97–1.01	0.280
Resection weight	1.00	1.00–1.01	0.061
Depression	1.21	0.79–2.38	0.818
Pulmonary disease	1.18	0.13–10.64	0.883
Blood loss	1.00	0.99–1.00	0.647
Hypertension	2.83	0.64–12.58	0.172
TXA	0.35	0.60–3.02	0.082
Herbal supplement	2.27	0.44–11.69	0.327

## Discussion

This study demonstrates that intraoperative tranexamic acid (TXA) significantly reduces the incidence of postoperative hematomas requiring surgical intervention following reduction mammaplasty. TXA use was associated with a more than twofold reduction in hematoma incidence, consistent with previous studies reporting decreased risk of postoperative hematomas [2, 11, 22, 25, 26]. Prior research has also suggested that TXA may reduce postoperative drainage volume and shorten hospital length of stay.

Given that reduction mammaplasty is a commonly performed procedure in plastic surgery, identifying strategies to minimize complications is crucial for both patients and surgeons [13]. High body mass index (BMI), smoking, and diabetes have been previously identified as risk factors for increased postoperative complications [13]. These factors are routinely addressed preoperatively through smoking cessation programs, BMI thresholds, and optimization of glycaemic control in patients with diabetes. The routine use of TXA may represent a promising adjunctive strategy to further reduce postoperative complications and enhance the safety of reduction mammaplasty.

The two study groups were comparable (Table 1), with no statistically significant differences in baseline characteristics, suggesting that observed differences in outcomes are unlikely to be attributable to confounding patient factors. The mean operative time was significantly longer in the TXA-exposed group (133.1 minutes) compared with the unexposed group (122.5 minutes; *p* < 0.001). This likely reflects case complexity and surgeon selection patterns rather than prolonged hemostasis, as TXA was more frequently administered in anticipated higher-risk cases. Although multivariable analysis adjusted for measurable confounders, residual selection bias inherent to the retrospective design cannot be excluded.

The longer operative time may also reflect the fact that, in many cases, TXA was applied in response to excessive intraoperative bleeding. Meticulous hemostasis requires additional surgical time, and TXA administration may contribute marginally to overall procedure duration. In contrast, Om et al. [22] reported no significant difference in operative time between TXA and control groups (124.9 vs. 123.7 minutes; *p* = 0.87).

Notably, mean intraoperative blood loss was higher in the TXA-exposed group (263.8 mL) than in the unexposed group (229.5 mL). This may reflect the timing of TXA administration, which was typically at anaesthetic induction or initiated by the surgeon in response to observed bleeding. Similarly, Om et al. [22] reported no significant difference in blood loss between groups (74.0 vs. 73.0 mL; *p* = 0.90).

Mean follow-up time was significantly shorter in the TXA-exposed group (3.2 months) compared with the unexposed group (5.0 months; *p* < 0.001), which may suggest that patients receiving TXA experienced faster recovery and required fewer prolonged follow-up visits. At our center, the standard follow-up period after reduction mammaplasty is one to three months, with additional visits typically reserved for patients experiencing postoperative complications. To our knowledge, no previous studies on TXA use in reduction mammaplasty have specifically reported follow-up duration, limiting direct comparisons.

The use of TXA in reduction mammaplasty has been investigated in multiple retrospective analyses [2, 11, 18, 22, 25]. However, only three independent double-blind randomized controlled trials (RCTs) have been conducted: Ausen et al. [26], Yao et al. [27], and Abboud et al. [28]. Ausen et al. performed a double-blind RCT on topical TXA in 28 patients (56 breasts), demonstrating a 39% reduction in drain fluid production [26]. Yao et al. conducted a double-blind RCT on topical TXA in 98 patients (196 breasts) and reported no reduction in postoperative hematoma rates [27]. Abboud et al. investigated topical TXA in reduction mammaplasty combined with liposuction and resection; as this study included combined procedures, it is not directly comparable to our cohort, which focused on resection-only reduction mammaplasty [28]. Overall, only two RCTs with fewer than 100 patients each have directly evaluated the effect of TXA on postoperative complications following reduction mammaplasty [26, 27].

Four meta-analyses have consistently demonstrated that TXA use is associated with a reduction in postoperative hematoma incidence [3, 12, 15, 29].

No substantial reduction in postoperative seroma rates was observed following TXA administration. While two studies reported a trend toward decreased seroma formation with TXA, the majority reported similar or slightly increased rates. Postoperative seroma formation remains a topic of debate, with various theories regarding its underlying mechanisms. Seroma prevention is largely attributed to meticulous surgical technique and postoperative management, suggesting that TXA may have a limited role in seroma reduction [2, 25, 26]. In our study, no differences in seroma incidence were observed between the TXA-exposed and unexposed cohorts, which may reflect the limited and inconsistent use of surgical drains at our institution.

In contrast, intraoperative blood loss measurements were consistently higher in the TXA cohort, likely reflecting clinical decision-making: surgeons may administer TXA (intravenously, topically, or both) when encountering apparent increased bleeding or anticipating a higher risk of haemorrhage.

No differences in surgical site infection (SSI) incidence were observed between cohorts. Similarly, four previous studies reported no significant differences in SSI risk following TXA administration [11, 18, 22, 25]. These findings are consistent with infection trends observed in other TXA-treated breast procedures [31–33]. Reported SSI incidence following breast reduction surgery varies widely, from 3 to 15%, influenced by institutional protocols, patient behaviours, and procedure type [13, 30].

We did not observe differences in overall complication rates between the TXA-exposed and unexposed cohorts. In contrast, previous studies reported an overall complication rate of 45% in the TXA group versus 57% in controls, corresponding to an absolute reduction of 11.9% with TXA use (*p* = 0.002) [12]. Discrepancies among studies may be explained by differences in inclusion criteria, patient demographics, surgical techniques, and definitions of complications. The relatively high complication rates in our cohort may limit the generalizability of our findings, suggesting that our study population had a higher baseline risk, which could amplify the apparent efficacy of TXA in reducing complications.

Given the antifibrinolytic properties of TXA, concerns have been raised regarding venous thromboembolism (VTE) as a potential adverse effect. Reassuringly, none of the previous studies assessing VTE incidence in TXA-treated patients reported postoperative thromboembolic events [3, 12, 15, 29]. These findings are consistent with prior research demonstrating minimal to no thrombotic risk associated with TXA administration [34, 35].

However, intravenous TXA has been associated with an increased risk of thromboembolic complications, particularly in patients with predisposing conditions. Topical or local administration represents a viable alternative, potentially minimizing systemic absorption and reducing the risk of thrombotic events, especially in patients with uncertain thrombotic risk [36, 37].

Dosing regimens varied across studies, ranging from single doses to administration combined with tumescent solution and epinephrine, which may have influenced observed outcomes. In breast surgery, the most commonly used doses are 1 g intravenously or 20–25 mg/mL topically, consistent with the regimen applied in our study [26, 38].

Our study demonstrated that TXA use in reduction mammaplasty was associated with more than a twofold reduction in postoperative hematomas requiring reoperation. This finding aligns with results reported in four independent meta-analyses by Liechti et al., Alhebshi et al., Fung et al., and Calpin et al. [3, 12, 15, 29], but differs from the double-blind randomized controlled trial conducted by Yao et al. [27]. Overall, TXA exposure was generally associated with lower rates of postoperative complications, particularly hematoma formation (Table 3). Variations in the definitions of postoperative hematoma, as well as potential biases in clinical assessment, may explain discrepancies in hematoma rates reported across previous studies. Nevertheless, the present analysis supports the effectiveness of TXA in reducing complications in breast surgery.

A major strength of this study is the large, single-center cohort of consecutive patients undergoing bilateral reduction mammaplasty, which, to the authors’ knowledge, represents the largest series reported in the literature evaluating TXA use in this context, including 656 patients (1,312 breasts). By comparison, the second largest study, conducted by Sipos et al. in 2023, included 415 patients (785 breasts) [11]. Comprehensive documentation of preoperative parameters allowed for detailed analysis of patient characteristics and potential confounders. Inclusion of all consecutive patients over a five-year period minimized selection bias and enhanced both transparency and reproducibility. Furthermore, the study reflects real-world clinical practice, as multiple board-certified surgeons performed the procedures, providing a heterogeneous and representative sample of surgical decision-making, operative techniques, and TXA administration strategies. The detailed capture of intraoperative variables, postoperative complications, and follow-up outcomes strengthens the validity of the findings and supports their applicability to routine clinical practice.

This study has several limitations. First, the retrospective design inherently limits causal inference and is subject to potential biases in data collection. Detection of postoperative hematomas was largely based on clinical assessment, which is inherently subjective; some surgeons may elect to evacuate even small hematomas, while others adopt a more conservative approach. Patient preoperative characteristics may also have influenced outcomes: in our cohort, TXA-exposed patients had a higher comorbidity burden and a greater proportion of smokers (Table 1), representing potential confounding factors. Data were collected from existing medical records originally documented for clinical, rather than research, purposes, which may be incomplete, inconsistent, or lack key variables. Documentation practices were not standardized, as multiple surgeons with varying recording habits contributed to the dataset, which could introduce measurement bias.

Intravenous TXA achieves systemic antifibrinolytic activity by competitively inhibiting plasminogen activation, whereas topical TXA provides localized clot stabilization with minimal systemic absorption. Topical administration may theoretically reduce thromboembolic risk while maintaining local hemostatic efficacy. However, due to heterogeneity in administration routes and limited subgroup power, our study was not designed to directly compare intravenous versus topical efficacy or evaluate dose-response relationships.

Furthermore, this single-center study was conducted at an academic teaching hospital, which may limit generalizability due to reduced population heterogeneity and potential systematic bias introduced by local clinical practices and surgeon preferences. Variations in surgical technique, instruments, intraoperative decision-making, and perioperative care may have influenced outcomes, including postoperative complication rates. The decision to administer TXA was based on individual surgeon preference, resulting in non-uniform use across patients and potential selection bias toward higher-risk cases. Additionally, follow-up duration varied between groups, which may have influenced detection of late complications or reoperations. Finally, although the cohort size was large, the absolute number of specific complications such as hematoma requiring reoperation remained relatively low, limiting the statistical power to detect small differences or rare adverse events.

Further prospective studies and randomized controlled trials are warranted to refine treatment protocols and establish evidence-based guidelines for TXA use in reduction mammaplasty. Future research should directly compare administration routes, particularly intravenous versus topical application, to provide clearer guidance on efficacy and safety. Additionally, studies investigating optimal dosing regimens, timing of administration, and sustained delivery strategies are needed to determine the safest and most effective TXA protocols. Research should also evaluate patient-centered outcomes, such as postoperative pain, recovery time, and quality of life, to better capture the clinical benefits of TXA beyond hematoma reduction. Subgroup analyses examining high-risk populations, such as patients with elevated BMI, smokers, or those with comorbidities, could help identify patient groups most likely to benefit from TXA. Finally, multicenter studies would improve generalizability by accounting for variations in surgical technique, institutional protocols, and surgeon experience, while also providing sufficient power to detect rare adverse events, including thromboembolic complications.

## Conclusion

The use of TXA in reduction mammaplasty was associated with a more than twofold reduction in the incidence of postoperative hematomas requiring reoperation. In this large retrospective cohort, TXA administration demonstrated a significant protective effect against hematoma formation, supporting its antifibrinolytic benefit as reported in prior studies. However, the surgeon-dependent and non-standardized administration limits causal inference, and variability in dosing, route, and patient selection should be considered when interpreting these findings.

As a simple, low-risk, and cost-effective intervention, TXA has the potential to substantially reduce postoperative bleeding and the associated need for reoperations. Prospective, randomized, and ideally multicenter studies are warranted to define optimal dosing strategies, compare intravenous versus topical administration, and identify patient populations most likely to benefit. Such studies would provide the evidence necessary to develop standardized, evidence-based clinical guidelines for TXA use in reduction mammaplasty, ultimately improving patient safety and surgical outcomes.
